# White matter and neurite morphology differ in psychogenic nonepileptic seizures

**DOI:** 10.1002/acn3.51198

**Published:** 2020-09-29

**Authors:** Adam M. Goodman, Jane B. Allendorfer, Andrew S. Blum, Mark S. Bolding, Stephen Correia, Lawrence W. Ver Hoef, Tyler E. Gaston, Leslie E. Grayson, Nina V. Kraguljac, Adrienne C. Lahti, Amber N. Martin, William S. Monroe, Noah S. Philip, Krista Tocco, Valerie Vogel, W. Curt LaFrance, Jerzy P. Szaflarski

**Affiliations:** ^1^ Department of Neurology and the UAB Epilepsy Center University of Alabama at Birmingham Birmingham Alabama USA; ^2^ Department of Neurology Rhode Island Hospital Providence Rhode Island USA; ^3^ Brown University Providence Rhode Island USA; ^4^ Department of Radiology University of Alabama at Birmingham Birmingham Alabama USA; ^5^ Department of Psychiatry and Human Behavior Alpert Medical School Brown University Rhode Island Hospital Providence Rhode Island USA; ^6^ Center for Neurorestoration and Neurotechnology Providence VA Medical Center Providence Rhode Island USA; ^7^ Birmingham VA Medical Center Birmingham Alabama USA; ^8^ Children’s of Alabama University of Alabama at Birmingham Birmingham Alabama USA; ^9^ Department of Psychiatry and Behavioral Neurobiology University of Alabama at Birmingham Birmingham Alabama USA; ^10^ Departments of Neurobiology and Neurosurgery University of Alabama at Birmingham Birmingham Alabama USA; ^11^ Department of Research Computing University of Alabama at Birmingham Birmingham Alabama USA; ^12^ Departments of Psychiatry and Neurology Rhode Island Hospital and Brown University Providence Rhode Island USA; ^13^ Comprehensive Neuroscience Center University of Alabama at Birmingham Birmingham Alabama USA

## Abstract

**Objective:**

To further evaluate the relationship between the clinical profiles and limbic and motor brain regions and their connecting pathways in psychogenic nonepileptic seizures (PNES). Neurite Orientation Dispersion and Density Indices (NODDI) multicompartment modeling was used to test the relationships between tissue alterations in patients with traumatic brain injury (TBI) and multiple psychiatric symptoms.

**Methods:**

The sample included participants with prior TBI (TBI; N = 37) but no PNES, and with TBI and PNES (TBI + PNES; N = 34). Participants completed 3T Siemens Prisma MRI high angular resolution imaging diffusion protocol. Statistical maps, including fractional anisotropy (FA), mean diffusivity (MD), neurite dispersion [orientation dispersion index (ODI)] and density [intracellular volume fraction (ICVF), and free water (i.e., isotropic) volume fraction (V‐ISO)] signal intensity, were generated for each participant. Linear mixed‐effects models identified clusters of between‐group differences in indices of white matter changes. Pearson’s r correlation tests assessed any relationship between signal intensity and psychiatric symptoms.

**Results:**

Compared to TBI, TBI + PNES revealed decreases in FA, ICVF, and V‐ISO and increases in MD for clusters within cingulum bundle, uncinate fasciculus, fornix/stria terminalis, and corticospinal tract pathways (cluster threshold α = 0.05). Indices of white matter changes for these clusters correlated with depressive, anxiety, PTSD, psychoticism, and somatization symptom severity (FDR threshold α = 0.05). A follow‐up within‐group analysis revealed that these correlations failed to reach the criteria for significance in the TBI + PNES group alone.

**Interpretation:**

The results expand support for the hypothesis that alterations in pathways comprising the specific PNES network correspond to patient profiles. These findings implicate myelin‐specific changes as possible contributors to PNES, thus introducing novel potential treatment targets.

## Introduction

Psychogenic nonepileptic seizures (PNES), (also referred to as dissociative or functional seizures), are a functional neurological symptom (conversion) disorder (FNSD) characterized by episodes resembling epileptic seizures or convulsions (DSM‐5 300.11; ICD‐10 F44.5) not associated with ictal discharges.^1^ While PNES are associated with underlying psychological conflicts or stressors and psychiatric comorbidities, many patients also report history of traumatic brain injury (TBI).^2^ Patients with FNDs, including PNES, often report increased depression, anxiety, and posttraumatic stress symptom severity,^3^ and their clinical outcomes are often linked to comorbid anxiety and mood disorders.^4^, ^5^ Current treatments for FNSDs include evidence‐based psychotherapies^1^, ^6^ but the neurobiological mechanisms for symptom improvement with therapy are not well understood.

There is an emerging literature that patients with PNES exhibit alterations in brain structure and function observed in other FNSDs via disruption of normal emotion and motor function processes.^7^ Recent studies of PNES and other FNSDs have begun to elucidate the neural basis for these disruptions by examining corresponding changes in volume and function of brain regions,^8^, ^9^, ^10^, ^11^ AUTHOR: Please check and confirm whether the funding information is correct^8^, ^9^, ^10^, ^11^ yet changes in the structural connections of these brain networks remains less well understood.^12^ Determining network functionality and connectivity that underlies PNES may increase our ability to develop new therapies to target‐specific parts of the network that yield greater treatment efficacy.

Structural imaging approaches, such as diffusion magnetic resonance imaging (dMRI), may be used to identify the implicated neural networks. DMRI is typically used to assess the integrity of the white matter (WM) pathways via diffusion metrics, including fractional anisotropy (FA), mean diffusivity (MD), and deterministic tractography metrics, including fiber bundle density and length. To our knowledge, only four preliminary PNES studies have demonstrated standard DTI measure alterations within sensorimotor, default‐mode, attention, and emotion regulation functional networks.^13^, ^14^, ^15^, ^16^ More specifically, PNES have been linked to decreased FA and asymmetry of fiber bundle indices within the uncinate fasciculus pathway (i.e., emotion regulation),^13^, ^14^ widespread decreases in FA and increases in MD,^16^ and reduced small‐worldness (i.e., shortest mean path‐length) among attention, sensorimotor, subcortical, and default‐mode networks.^15^ Likewise, other FNSD (i.e., functional dystonia), have been linked to global WM disconnection affecting main sensorimotor and emotional control circuits,^17^ whereas FA decreases within stria terminalis/fornix, medial forebrain bundle, extreme capsule, uncinate fasciculus, cingulum bundle, corpus callosum, and striatal‐postcentral gyrus projections have been linked to mixed FNSDs.^18^

Recently, specialized dMRI sequences known as high‐angular resolution diffusion imaging (HARDI) have been made available to extend traditional assessment of WM integrity by further modeling distinct neuronal compartments, or neurites. Specifically, the neurite orientation dispersion and density indices (NODDI) toolbox^19^ can be utilized to assess neurite dispersion [orientation dispersion index (ODI)] and density [intracellular volume fraction (ICVF), and isotropic‐free water volume fraction (V‐ISO)]. The statistical maps generated by the NODDI toolbox provide greater specificity for alterations and additional WM pathophysiologic information compared to traditional dMRI indices.

In this study, we utilized advanced NODDI analysis methods to evaluate the WM pathways within the PNES network model and to extend our understanding of PNES as a network disorder. Given that the majority of patients with PNES report one or more prior TBI,^2^ comparing PNES to a control group without prior TBI would not adequately control for the likelihood of TBI in PNES and potentially produce collinearity among factors of TBI and PNES occurrence between groups. Furthermore, emotion networks are already inherently changed by the virtue of physical neurotrauma, and emotional processing is altered by the traumatic event(s).^20^ Accordingly, TBI was selected as a model in the current investigation to better approximate the PNES population for studying networks involved in the development and maintenance of PNES. We hypothesized that WM integrity and neurite morphology assessments would demonstrate distinct alterations in the TBI with PNES group (TBI + PNES) compared to TBI without PNES group (TBI). Specifically, we assessed alterations in FA, MD, ODI, ICVF, and V‐ISO indices between groups. Based on prior literature, we expected to find group differences within WM pathways that connect limbic and motor regions of the brain, and that differences in DTI and NODDI measures would correspond to distinct mental health profiles in PNES, including depressive, anxious, posttraumatic, psychoticism, and somatization symptoms. Based on prior literature, we hypothesized that increased depressive, anxious, posttraumatic and psychoticism symptoms of PNES would correspond to group differences within the uncinate fasciculus, fornix/stria terminalis, cingulum, whereas increased somatization symptoms would correspond to group differences within the corticospinal tract.^6^, ^8^, ^9^, ^10^, ^11^, ^21^, ^22^, ^23^, ^24^

## Methods

### Participants

Seventy‐one participants were recruited prospectively from three sites [(1) Rhode Island Hospital, RIH; (2) Providence Veterans Affairs Medical Center, PVAMC; and (3) University of Alabama at Birmingham, UAB]. Participants were separated into two groups consisting of 37 TBI and 34 TBI + PNES participants. Diagnosis of PNES was established in all participants according to recommendations of the International League Against Epilepsy (e.g., video EEG confirmed PNES).^25^ TBI was established by history and medical record review, along with the TBI‐screening questionnaire.^26^ History of TBI was reported by participants, including the number experienced, as well as the severity and duration since each TBI. The TBI + PNES participants also reported age of PNES onset. Participants were not excluded from the study based on visible lesions nor depending on whether consciousness was preserved in the temporal period surrounding the TBI. All protocols for this study received prior approval by the Institutional Review Boards of the participating institutions. All participants provided informed consent prior to participation in the study.

### Psychiatric and behavioral assessments

After consenting and prior to MRI scanning, all participants completed a series of clinical questionnaires assessing for commonly reported psychiatric and behavioral PNES and TBI comorbidities. Scales were selected based on prior literature on FNSDs demonstrating sensitivity to greater symptom severity compared to healthy controls, as well as relationships between limbic and motor brain regions structure and/or function.^3^, ^8^, ^9^, ^10^, ^11^, ^21^, ^27^ Independent samples t‐tests were used to compare TBI and TBI + PNES groups for depressive (Beck Depression Inventory‐II, BDI‐II),^28^ anxious (Beck Anxiety Inventory, BAI),^29^ posttraumatic stress (PTSD Checklist‐Specific version, PCL‐S),^30^ psychoticism (Symptom Checklist‐90, SCL‐90 PSY), somatization (SCL‐90 SOM)^31^ symptoms, and symptom severity (Global Assessment of Functioning).^32^ Corrected degrees of freedom were used for any comparison that violated assumptions for homogeneity of variance between groups.

### MRI parameters and analysis

**Figure 1:**
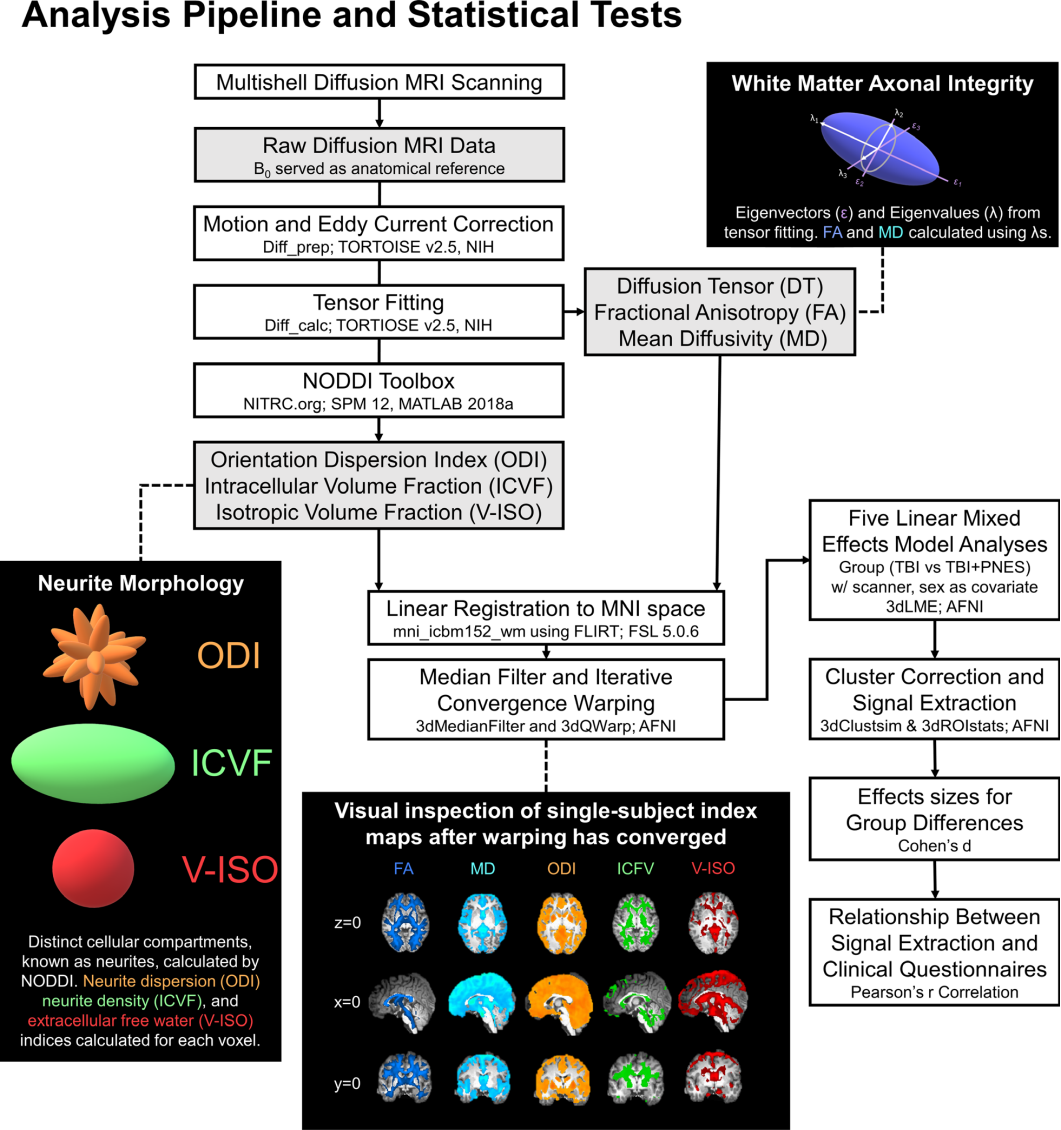
Schematic depicting the diffusion imaging analysis pipeline, modeling examples for DTI and NODDI indices, postwarping visual inspection example, and statistical tests performed.

After prescreening, consenting, and initial assessments, participants completed HARDI protocols performed on two (RIH and UAB) 3 Tesla Prisma scanners (Siemens Healthcare, Erlangen, Germany) using a 64‐channel head coil. Acquisition protocols were carefully harmonized *a priori* in this study. All scanner firmware, software, and hardware upgrades, if performed, were synchronized between sites. A multishell diffusion scheme (humanconnectome.org) with b‐values of 1500 and 3000 s/mm^2^ (47 and 46 directions, respectively) and 6 B0 images were acquired using a single‐phase encoding direction (anterior‐to‐posterior) with the following protocol: TR = 3230 ms, TE = 89.2 ms, FOV = 21 × 21 cm, flip angle 78 degrees, multiband factor 4, 1.5 mm isotropic voxels. At both sites, auto‐alignment localizers reduced variability in subject positioning using anatomical landmarks to direct placement of the FOV. Following data acquisition, a pipeline utilizing standard image processing software was used to preprocess, model, and compare the data (Figure 1). Each dataset was first corrected for motion, eddy currents and susceptibility artifacts, and rotation of gradient tables by selecting the standard option to include these adjustments in TORTOISE (v2.5.2b, nih.gov; DIFF_PREP). Briefly, diffusion tensor imaging (DTI) and NODDI metrics were estimated after preprocessing using TORTOISE (v2.5.2b, nih.gov; DIFF_CALC with linear fitting algorithm) and the NODDI toolbox (v1.01, nitrc.org)^19^ in MATLAB R2018a (mathworks.com; MA, USA). Resulting statistical maps from this preprocessing pipeline produced five separate whole‐brain maps for each subject, including FA, MD, ODI, ICVF, and V‐ISO. To smooth and then spatially normalize diffusion images to the McConnell Brain Imaging Centre standard (ICBM 2009a Nonlinear Symmetric WM Template), AFNI (afni.nimh.nih.gov) algorithms were used to perform spatial smoothing (3dMedianFilter) to a 2 voxel (3mm) neighborhood radius on each participant’s images before an optimized nonlinear image registration (3dQwarp) performed an iterative refinement on FA maps with a convergence criterion at each patch level to better resolve artifacts (final patch size of 3 mm isotropic). The resulting transformation matrix derived from warping the FA statistical map was then applied to warp the four remaining statistical maps for each subject (3dNwarpApply). For each subject, the five warped whole‐brain statistical maps underwent visual inspection to ensure the validity of each statistical map and successful warping onto the MNI standard template (Figure 1). Specifically, each statistical map was inspected for spatial overlap between voxels with relatively high intensity that correspond to respective tissue regions of the MNI anatomical standard template. In other words, registration of V‐ISO and MD were validated with high concordance to CSF in the ventricles/interhemispheric fissure, ODI was validated with high concordance to cortical gray matter regions, and ICVF and FA were validated with high concordance to white matter regions of the template.

Whole brain voxel‐wise group differences (TBI vs TBI + PNES) were assessed using AFNI's 3dLME for each of the five diffusion maps. Biweekly quality assurance was performed at both UAB and RIH scanners using the multicenter collaborative fMRI research project (FIRST‐BIRN) quality assurance protocol^33^ and confirmed that Signal‐to‐Noise Ratio (SNR), Signal‐to‐Fluctuation‐Noise Ratio (SFNR), signal fluctuations, and signal drift were stable throughout the study. All 3dLMEs included an intercept to remove variability as a factor of scanner site (UAB, RIH) and sex (male, female). A WM mask derived from the ICBM 2009a WM Template, restricted 3dLME analyses to anatomically based WM boundaries in order to lower the overall number comparisons and reduce family wise error (FWE) rates. Specifically, based on prior literature, we hypothesized there would be group differences within the uncinate fasciculus (UF; prefrontal cortex‐hippocampus‐amygdala), fornix/stria terminalis (FST; hippocampus‐amygdala‐hypothalamus), cingulum (cingulate cortex‐dorsal/ventral prefrontal cortex), and corticospinal tract (pre/post central gyrus‐spinal cord).^6^, ^8^, ^9^, ^10^, ^11^, ^21^, ^22^, ^23^ In order to further reduce the likelihood of type‐I error, volume extent thresholds (mm^3^) were calculated using AFNI’s 3dClustSim based on an algorithm that uses randomization/permutation simulation to produce 10 000 iterations of noise only generated t‐tests and to determine the global cluster‐level threshold values for each of the five separate diffusion measures. Only clusters identified by 3dLMEs exceeding 443 mm^3^ for FA, 460 mm^3^ for MD, 437 mm^3^ for ODI, 548 mm^3^ for ICVF, and 612 mm^3^ for V‐ISO were considered statistically significant (uncorrected *P* < 0.01, cluster threshold α = 0.05). Signal extractions (3dROIstats) were performed for clusters identified by the 3dLMEs that included *a priori* pathways of interest within UF, FST, cingulum (JHU‐DTI WM labels),^34^ and corticospinal tract (JHU‐DTI tract probability map)^35^ regions. To interpret any clusters of significant group differences identified by the 3dLMEs, mean signal and Cohen’s d estimate of effect size determined the direction and strength of effects.

### Brain and behavior comparisons

To better characterize the clinical implications of observed group differences in WM, Pearson’s r correlations assessed the relationship between each cluster’s mean signal with scores on BDI‐II, BAI, PCL‐S, SCL‐90 PSY, and SCL‐90 SOM scales across both the TBI and TBI + PNES groups. To reduce the type‐I error rate from multiple comparisons, a false discovery rate corrected threshold (FDR threshold α = 0.05, two‐tailed) was used to determine any significant correlations. Additional follow‐up Pearson’s r correlation tests (FDR threshold α = 0.05, two‐tailed) assessed the relationship between each cluster’s mean signal with scores on BDI‐II, BAI, PCL‐S, SCL‐90 PSY, SCL‐90 SOM, symptom severity (GAF^36^), and PNES duration (age of onset subtracted from current age) assessments for only the TBI + PNES group to contextualize potentially specific relevance of the psychiatric variables in PNES. To assess the potential role of age effects, all signal extractions were compared to age (years) using Pearson’s r correlation tests (uncorrected α = 0.05, two‐tailed).

## Results

### Participant demographics and TBI history

Results for all statistical comparisons (α = 0.05, two‐tailed) of demographic factors and TBI history between TBI and TBI + PNES groups are reported in Table 1. There were no differences between the groups with respect to age, and as expected based on PNES epidemiology,^37^ the proportion of female participants was greater in the TBI + PNES group. Likewise, the number of TBIs, time since TBI, TBI severity, TBI hemispheric laterality, and TBI lobe did not differ between groups (all *P* > 0.05; Table 1).

**Table 1:** Demographics and TBI, Psychiatric, and Behavioral Assessments by Groups.

	Groups		Stat	*P*‐value
	TBI	TBI + PNES		
Demographics and TBI history				
Total samples	n = 37	n = 34	‐	‐
Birmingham, AL enrollees	n = 9	n = 15	‐	‐
Providence, RI enrollees	n = 26	n = 19	‐	‐
Years of Age	39.7 [10.9]	36.7 [11.8]	*t* = 1.2	0.27
Sex (Female)	n = 16	n = 24	*χ* ^2^ = 4.8	<0.05*
PNES duration (years)		5.73 [8.88]		
Number of TBIs	4.7 [4.7]	5.5 [8.6]	*t *= −0.4	0.66
Duration since TBI (months)	91.8 [103.8]	107.3 [130.7]	*t *= *−0.6*	0.58
TBI severity	mild; n = 31	mild; n = 28	*χ* ^2^ *= *0.3	0.87
	moderate; n = 3	moderate; n = 3		
	severe; n = 1	severe: n = 1		
	unknown; n = 2	unknown; n = 2		
TBI hemispheric laterality	left; n = 5	left; n = 3	*χ* ^2^ *= *0.9	0.82
	right; n = 8	right; n = 8		
	bilateral; n = 16	bilateral; n = 13		
	unknown; n = 8	unknown; n = 10		
TBI lobe(s)	frontal; n = 11	frontal; n = 13	*χ* ^2^ * = *3.5	0.63
	parietal; n = 4	parietal; n = 2		
	occipital; n = 6	occipital; n = 4		
	temporal; n = 2	temporal; n = 0		
	multiple; n = 8	multiple; n = 7		
	unknown; n = 6	unknown; n = 8		
Psychiatric and behavioral assessments				
Current Mood disorder (yes)	n = 14	n = 21	*χ^2^ = *1.3	0.25
Current Anxiety disorder (yes)	n = 14	n = 19	*χ^2^ = *0.5	0.49
GAF	76.24 [14.62]	53.52 [7.87]	*t = *8.2	<0.001*
BDI‐II	12.9 [13.5]	25.6 [13.1]	*t* = −4.0	<0.001*
BAI	12.3 [13.3]	27.8 [11.1]	*t* = −5.3	<0.001*
PCL‐S	33.9 [17.0]	50.4 ]14.9]	*t* = −4.2	<0.001*
SCL‐90 PSY	4.7 [6.1]	6.8 [6.8]	*t* = −1.4	0.18
SCL‐90 SOM	8.8 [8.3]	18.2 [10.3]	*t* = −4.2	<0.001*

Data for TBI versus TBI + PNES patients reported as mean [SD], except for Sample Size, Sex, Prior Mood, and Anxiety disorders, TBI severity, TBI hemispheric laterality, and TBI lobe, which are reported as counts (n). Chi‐squared test (χ^2^) tested the null hypothesis that the proportions for counts did not differ between groups. All other comparisons were tested using an independent samples *t*‐test (t). The between group test statistic (Stat) and *P*‐value (*P*) are presented.

* Indicates a comparison that reached statistical significance (α = 0.05, two‐tailed).

### Psychiatric and behavioral assessments

Results for all statistical comparisons (α = 0.05, two‐tailed) of psychiatric and behavioral assessments between TBI and TBI + PNES groups are reported in Table 1. There were no differences between TBI and TBI + PNES groups with respect to prior psychiatric comorbidities or psychoticism scale symptoms, and as expected based on PNES epidemiology,^3^, ^27^ depressive, anxious, posttraumatic stress, and somatization symptoms were greater in the TBI + PNES group (Table 1).

### MRI analysis

**Figure 2:**
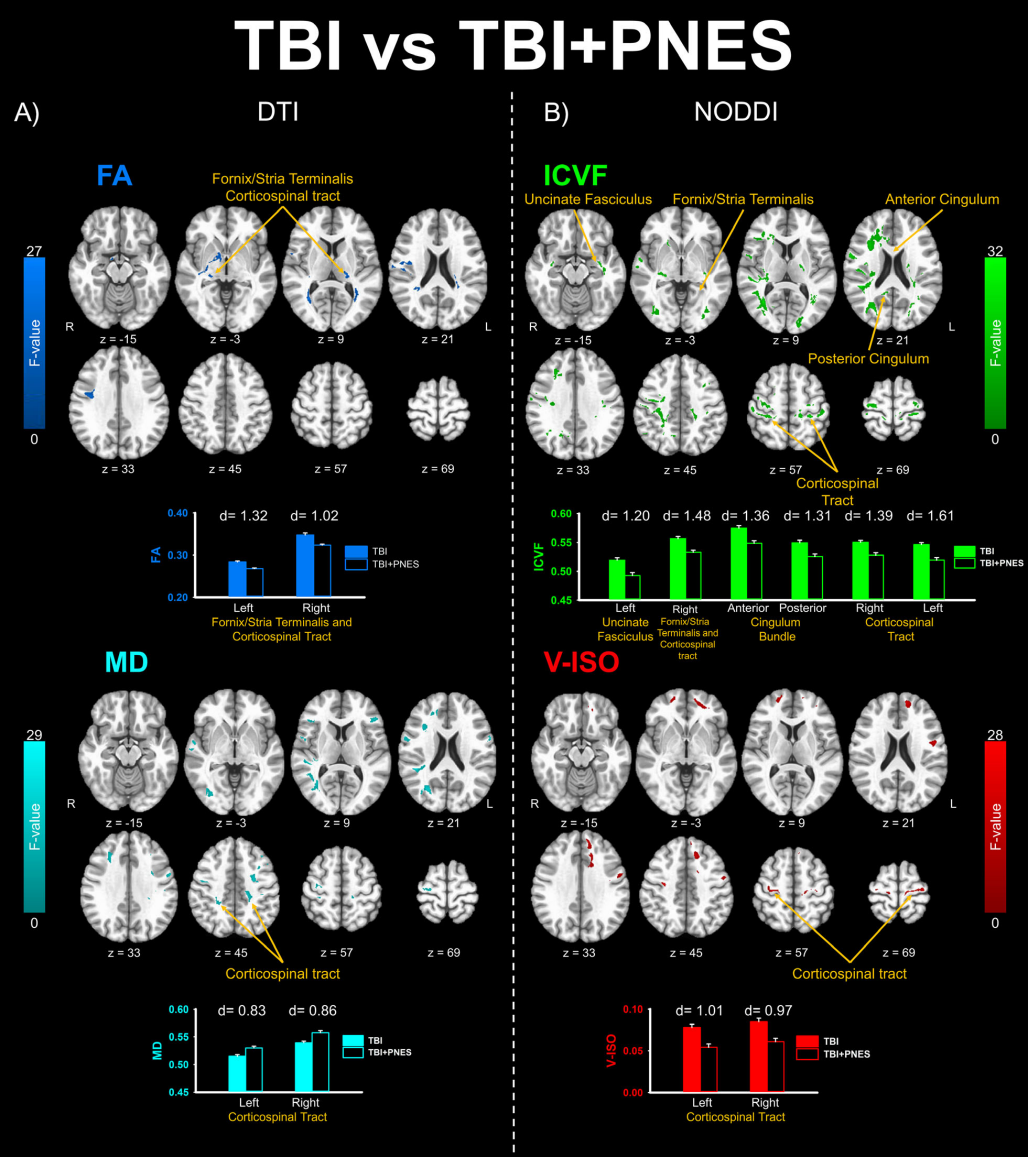
Results from the five linear mixed effects analysis comparing TBI versus TBI + PNES that survived the cluster correction volume threshold (uncorrected *P* < 0.01, cluster threshold α = 0.05). Clusters that survived group‐level analysis for dMRI‐based statistical maps (FA, Fractional Anisotropy; MD, Mean Diffusivity) appear in the left panel (A) and for NODDI‐based statistical maps (ICVF, Intracellular Volume Fraction, V‐ISO, Isotropic Volume Fraction) appear in the right panel (B). No clusters survived group‐level analysis for ODI measures. Volume extent thresholds (Monte Carlo simulation) were determined separately for FA (443mm^3^); MD (460mm^3^); ODI (437mm^3^); ICVF (548mm^3^); and V‐ISO (612 mm^3^) based on whole‐brain spatial noise distributions for each index. Clusters that included pathways identified as a priori tracts of interest are identified with yellow arrows and labels. Images appear in radiological view (R‐>L). In order to interpret the direction of statistically significant group differences identified by the 3dLMEs, cluster means are plotted for each group below statistical maps. The strength of these group differences was determined by comparing cluster‐wise signal mean compared between groups (TBI vs. TBI + PNES) using Cohen’s d estimate of effects size.

The results of the 3dLME analyses that compared DTI and NODDI indices between TBI and TBI + PNES groups, controlling for variability due to scanner site and sex, (corrected α = 0.05, two‐tailed) are reported in Figure 2. No clusters survived the correction for multiple comparisons in the 3dLME for group differences in ODI. Figure 2 (below each statistical map) shows the signal extraction means and Cohen’s d estimates of effect size derived from clusters identified by 3dLMEs within the a priori pathways of interest (UF, FST, cingulum, and corticospinal tract). Mean FA values were higher in the TBI than TBI + PNES groups for clusters within bilateral corticospinal tract and FST. Mean ICVF values were higher in the TBI than TBI + PNES groups for clusters within the right FST, left UF, bilateral corticospinal tract, and right cingulum pathways. Mean V‐ISO values were higher in the TBI than TBI + PNES groups for clusters within bilateral corticospinal tract pathways. Mean MD values were lower in the TBI than TBI + PNES groups for a cluster within bilateral corticospinal tract pathways.

### Brain and behavior comparison results

**Figure 3:**
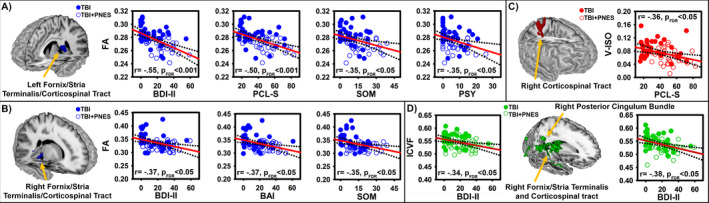
Depiction of significant linear relationships between mean signal comparisons within each of the 12 clusters (Figure 2), identified by tract and area within tracts, compared (Pearson’s r) against mood (BDI‐II,), anxiety (BAI), PTSD (PCL‐S), psychoticism (SCL‐90 PSY), and somatization (SCL‐90 SOM) symptom severity. Correlations were considered significant based on a false discovery rate (FDR) corrected threshold (FDR adjusted *P* < 0.05, two‐tailed). Strength and direction of correlations are indicated by r‐value and corresponding FDR adjusted *P*‐value (p_FDR_) within each scatterplot. Significant correlations with symptom severity were found for FA within the left (A) and right (B) fornix/stria terminalis/corticospinal tract pathways, for V‐ISO within the right corticospinal tract pathway (C), and for ICVF within the right fornix/stria terminalis/corticospinal tract pathways (D; left) and right posterior cingulum bundle pathway (D; right). All remaining correlations failed to reach the criteria for significance. Least squares regression lines (red line) and 95% confidence interval bands (dotted lines) were fitted to visually depict trend directions and variability across comparisons. Correlations, least‐squares regression lines, and confidence interval bands were calculated by collapsing TBI (closed circles) and TBI + PNES (open circles) groups. Follow‐up within‐group analyses revealed that these correlations failed to reach the criteria for significance among TBI or TBI + PNES group alone.

Figure 3 reports the results of the Pearson’s r (FDR corrected α = 0.05, two‐tailed) correlation tests that compared each of the five psychiatric assessments to mean signal values extracted from each of 12 clusters identified by the 3dLMEs that fell within the a priori pathways of interest (n = 60 comparisons). As bilateral FA signal decreased, there was a corresponding increase in values across all five measures (BDI‐II, BAI, PCL‐S, SCL‐90 PSY, and SCL‐90 SOM; Figure 3A‐B). V‐ISO values decreased within the right corticospinal tract with a corresponding increase in PCL‐S values (Figure 3C). ICVF values decreased within the right FST/corticospinal tract (Figure 3D, left) and posterior cingulum (Figure 3D, right) with a corresponding increase in BDI‐II values. All remaining Pearson’s r correlations for relationships between cluster mean signal values and psychiatric and behavioral assessments failed to reach criteria for significance. Within group post hoc results of all follow‐up Pearson’s r correlation tests (FDR corrected α = 0.05, two‐tailed) that compared each of the five psychiatric assessments, GAF, and PNES duration to mean signal values extracted from each of 12 clusters identified by the 3dLMEs for the TBI + PNES only group failed to reach criterial for significance. Likewise, the results of all Pearson’s r correlation tests (uncorrected α = 0.05, two‐tailed) that compared mean signal values extracted from each of 12 clusters identified by the 3dLMEs to age (years) failed to reach criteria for significance.

## Discussion

This prospective study assessed the hypothesis that PNES in patients with TBI can be conceptualized as a brain network disorder in which mental health symptom expression varies according to alterations in specific motor and limbic regional PNES networks.^7^ This hypothesis was formulated based on prior literature demonstrating that changes in structure and function of motor and limbic regions are associated with symptom expression in PNES^8^, ^9^, ^10^, ^11^, ^21^ and that PNES are associated with decreased WM integrity within neural projections that connect these brain regions.^13^, ^14^, ^15^ To this end, FNSDs including PNES have been conceptualized as a network brain disorder, rather than a focal deficit, in which patient profiles vary, depending on how limbic and motor regions that comprise the specific FNSD network are altered.^7^ While these recent volumetric and functional activation studies provide an important foundation for identifying the specific brain regions and networks involved in FNSDs including PNES, yet changes in the structural connections of these brain networks remain less well understood.

Preliminary studies have linked standard DTI measure (FA and fiber bundle) alterations to PNES within sensorimotor, default‐mode, attention, and emotion regulation functional networks^13^, ^14^, ^15^ and are consistent with findings that WM integrity is decreased within limbic and motor functional networks and associated with worsened psychiatric symptoms in mixed FNSDs.^18^ However, NODDI analysis further extends the PNES network model by providing a more specific assessment of tissue. Specifically, DTI analysis provides FA and MD statistical maps that serve as indices of WM integrity. As FA decreases and MD increases, there are corresponding decreases in nonspecific WM integrity and microstructure that may be affected by one or more tissue alterations that include, for example, axonal dispersion, density, or injury.^38^ Alternatively, NODDI analysis relates diffusion data to geometric models (Figure 1) providing a more targeted and specific assessment of tissue microstructure. Specifically, NODDI statistical maps separately index: 1) dispersion of crossing fiber orientations with increasing ODI values; 2) dense fiber bundling and myelination with increasing ICVF values; and 3) demyelination and/or edema with increasing V‐ISO values.^19^, ^39^, ^40^, ^41^ Unlike ODI and ICVF measures that have been histologically confirmed as a proxy of neurite dispersion and myelination,^39^ ex vivo histological validation of V‐ISO as a proxy of edema is not currently feasible due to the active physiological nature of this process^42^ or dehydration during sample preparation.^40^ Thus, microstructure changes that lead to fluctuation in V‐ISO measures within white matter regions may more broadly include neurodegenerative progression, including edema and/or axonal degeneration related to the myelin sheath.^43^

Despite similar severity of the TBI between groups, the TBI + PNES group had greater depressive, anxious, posttraumatic stress, psychoticism, and somatization symptoms compared to the TBI without PNES group. Furthermore, we observed an association between increasing symptoms of depression, anxiety, PTSD, psychoticism, and somatization symptom severity and decreasing WM integrity (i.e., FA), myelination (i.e., ICVF), and free water (i.e., V‐ISO) within pathways connecting motor and limbic networks. Although similar WM changes within limbic and motor networks have been previously identified in patients with TBI and psychiatric conditions^41^, ^44^, ^45^, this study extends these changes to PNES and highlights several key differences that are discussed below. By comparing individual differences in mental health symptom severity to differences in NODDI indices in TBI + PNES compared to TBI, we provide new evidence that suggests specific tissue microstructure alterations within motor and limbic WM pathways in TBI can be associated with the occurrence of mental health symptom expression in PNES.

In this study, PNES are associated with decreases in WM integrity within pathways connecting motor and emotion networks. Specifically, we found decreased FA values in TBI + PNES compared to TBI within bilateral FST and corticospinal tract. Amygdala and hippocampal regulation of the autonomic and endocrine response to stress^46^ critically involves projections through FST on to the hypothalamus.^34^ During processing of stressful information, hippocampal disinhibition serves as a release mechanism triggering the hypothalamic pituitary adrenal (HPA) axis and release of glucocorticoids in response to stress.^47^ Thus, decreased WM integrity within the fornix may be related to hippocampal hyporeactivty to stress in PNES.^24^ In general, increased glucocorticoid reactivity to stressors is associated with psychological vulnerability and psychosocial stress.^48^ The association between decreased FA within these clusters and increased mood, anxiety, PTSD, psychoticism, and somatization symptoms suggests that overlapping disruption of the corticospinal tract and FST may be associated with the diversity of comorbid mental health symptoms in PNES.

In this study, we observed reduced free water (i.e., V‐ISO) measures within corticospinal tract pathways among the PNES group that related to PTSD symptom severity. Although the biological specificity of increased free water remains unclear^40^, ^42^, ^43^, other studies report V‐ISO changes related to vasogenic edema^49^ or mylenation,^50^ which could be associated with chronic phase TBI. Given the lack of prior validation for neurobiological specificity for V‐ISO measures and that this study did not assess additional markers edema within WM pathways, future studies are needed to directly investigate any potential dynamic relationships between free‐water and posttraumatic stress symptoms in PNES.

Compared to TBI, TBI + PNES were associated with decreased ICVF within FST, UF, cingulum, and corticospinal tract WM pathways. These results extend prior studies on alterations in WM integrity within these networks for PNES,^13^, ^14^, ^15^ by implicating specific WM tissue microstructure (i.e., myelin). Additionally, decreases in ICVF within FST, corticospinal tract, and cingulum pathways were correlated with increased depressive symptom expression. Prior studies have reported that hyper‐functional connectivity between medial prefrontal cortex‐hippocampus‐posterior cingulate is linked to depressive mood symptoms.^51^, ^52^ Furthermore, another recent study found that changes in task‐elicited anterior cingulate/paracingulate activation was associated with improvement in functional tremor severity and depressive symptoms after cognitive behavioral therapy (CBT).^6^ Accordingly, myelination differences within cingulum and FST pathways may play an important role in the expression of depressive symptoms associated with PNES.

We hypothesized that the degree of changes in psychiatric symptoms and the degree of changes in white matter associated with PNES are related. This combination of groups in the analysis allows for modeling brain and behavior relationships to assess potential pathophysiological mechanisms that exist on a continuum and at both the clinical and subclinical level. Alternatively, post hoc follow‐up analysis that assessed only the TBI + PNES group was designed to contextualize the specificity of these relationships to PNES. The results of this follow‐up analysis failed to yield any significant relationships for symptom specificity. It is likely that the reason significant correlations were not observed for only TBI + PNES comparisons is that this restriction did not capture the full gamut of these relationships, given the relatively high and truncated psychiatric symptom severity in the PNES group. The results from including both groups in the correlation tests, in contrast, suggest that both mental health symptom severity and white matter differences for TBI + PNES vary from TBI in degrees, rather than in kind. We take the brain and behavior comparisons and post hoc comparisons results in this study to suggest that the increased expression of psychiatric symptoms is linked to more severe changes in white matter pathophysiology, rather than distinct relationships between these factors in PNES. Future studies may increase the understanding of such relationships by utilizing an independent within‐group analysis to assess white matter changes that vary with PNES symptom expression.

Several limitations should be considered when interpreting our findings. Our diffusion MR data acquisition was acquired using a single‐anterior‐to‐posterior phase encoding direction sequence; however, implementation of a complimentary reverse encoding direction sequence may improve susceptibility distortions that can degrade diffusion MRI data quality. Unlike tractography analysis, our voxel wise approach and hypotheses assessed white matter changes within dorsal and subcortical JHU white matter atlas regions. Although we did not assess areas that are highly susceptible to distortion when acquired in the anterior‐to‐posterior direction, such as cerebral cortex within anterior frontal and posterior occipital lobes, it is important to note that different phase encoding directions may still produce slightly different results^53^ when interpreting the results of this study in the context of the literature. Tractography analysis (i.e., changes in fiber bundle density and length) of NODDI metrics was not conducted in this study but may provide additional knowledge in future investigations of white matter differences in PNES and their role in symptom expression. While our sample size is moderate, this issue is mitigated by multisite recruitment and robust sample size compared to prior neuroimaging studies of PNES.^13^, ^14^, ^15^

Another limitation involves comparison group(s). Future studies may consider including a sample with epileptic seizures or mixed PNES and epilepsy comparison group in order to assess potential additive effects of epilepsy on white matter changes. Minimal prior investigation on white matter changes linked to TBI with epileptic seizures are a limitation for establishing the specificity of our findings in PNES compared to epilepsy. One human DTI study reported the ratio of FA values within TBI lesions compared to the corresponding contralateral MRI‐normal region and found significantly lower FA ratios in patients with epilepsy compared with those without epilepsy, but no significant difference in MD ratio for these same regions between groups.^54^ Thus, the lack of a comparison group in this study or prior literature investigating TBI and epileptic seizures limits our understanding of the specificity of white matter changes in PNES compared to epileptic seizures in chronic phase TBI. The absence of a healthy control group [i.e., without history of (non) epileptic seizures or TBI] comparison to contextualize the findings of this study; however, limits our understanding of how these white matter changes compare to a typical range for measures within these pathways. Furthermore, while this study was open to all severities of TBI, the majority of our sample is mild TBI; thus, how the findings generalize to moderate and severe TBI remains to be addressed in future studies. There was also an absence of a PNES group without TBI, other FNSDs, or related psychiatric diagnoses which may limit the generalizability of the findings. However, in agreement with the notion that PNES are part of the FNSD spectrum and an expression of the disruption in the different part of the FNSD network,^7^ we expect these findings to provide excellent springboard for further studies in FNSDs.

Lastly, this study utilized a limited subset of psychiatric comorbidity assessments, rather than implementing a broad exploratory evaluation that might include more targeted scales (e.g., Dissociative Experiences Scale)^55^, ^56^ or additional comorbidities (e.g., alexithymia)^57^ in PNES. The assessment scales utilized in this study were chosen *a priori* based on prior literature linking the specific comorbidities mental health states to neural function and structure, effectively reducing the potential for type‐I error rates inherent in exploratory studies. Future exploratory or targeted studies might implement such additional assessments to extend our assessment of relationships between alterations in WM tissue microstructure and psychopathology in PNES.

## Conclusions

By utilizing NODDI analysis to assess WM tissue microstructure in greater detail, this study provides evidence that PNES involves aberrant structural connectivity of brain networks. The current findings suggest pathophysiological relationships may exist between PNES symptoms and WM integrity and myelination, within cingulum, FST, UF, and corticospinal tract pathways. Future studies that utilize controlled clinical trials to assess changes in WM tissue microstructure that correspond to FNSD symptom improvement after psychotherapy^1^, ^6^ may increase our ability to develop therapies that target‐specific networks and nodes. Additionally, WM changes within motor and limbic networks that correspond with symptom improvement may lend support to the hypothesis that WM integrity and tissue microstructure may play a role in symptom profiles of patients with PNES.

## Author Contributions

WCL and JPS contributed conception and design of the study; AMG, JBA, MSB, ANM, VV, KT, and WSM contributed to acquisition and analysis of data; and AMG, JPS, JBA, ASB, SC, LWH, TEG, LRG, NVK, ACL, NSP, and WCL contributed to drafting of the manuscript.

## Role of the Sponsor

The Department of Defense had no role in the conduct of the study, manuscript preparation, or the decision to submit for publication. The views expressed in this article are those of the authors and do not necessarily reflect the position or policy of the Department of Veterans Affairs, Department of Defense, or the United States government.

## Additional Contributions

Frank Skidmore, MD, and Grayson Baird, PhD, provided consultation on study design and interpretation. Valencia Williams and Katlyn Jackson assisted with recruitment and data collections. Ravi Tripathi and Thomas Anthony provided technical support.

## Conflict of Interest

The authors do not report any disclosures of conflict of interest.
